# Supplementary material for: Crosstalk among WEE1 Kinase, AKT, and GSK3 in Nav1.2 Channelosome Regulation
Source: Int J Mol Sci. 2024 Jul 24;25(15):8069. doi: 10.3390/ijms25158069 (PMC11311446; doi:10.3390/ijms25158069)
Supplement: Supplementary file 1 [file ijms-25-08069-s001.zip › ijms-3058594-supplementary.docx]

Article

Crosstalk among WEE1 kinase, AKT, and GSK3 in Nav1.2 channelosome regulation

Aditya K. Singh *, Jully Singh, Nana A. Goode and Fernanda Laezza

^Department of Pharmacology & Toxicology, The University of Texas Medical Branch, Galveston, Texas, USA^

^* Corresponding author: Aditya K. Singh (email: adsingh@utmb.edu)^

**Supplementary materials**

Supplementary figures:


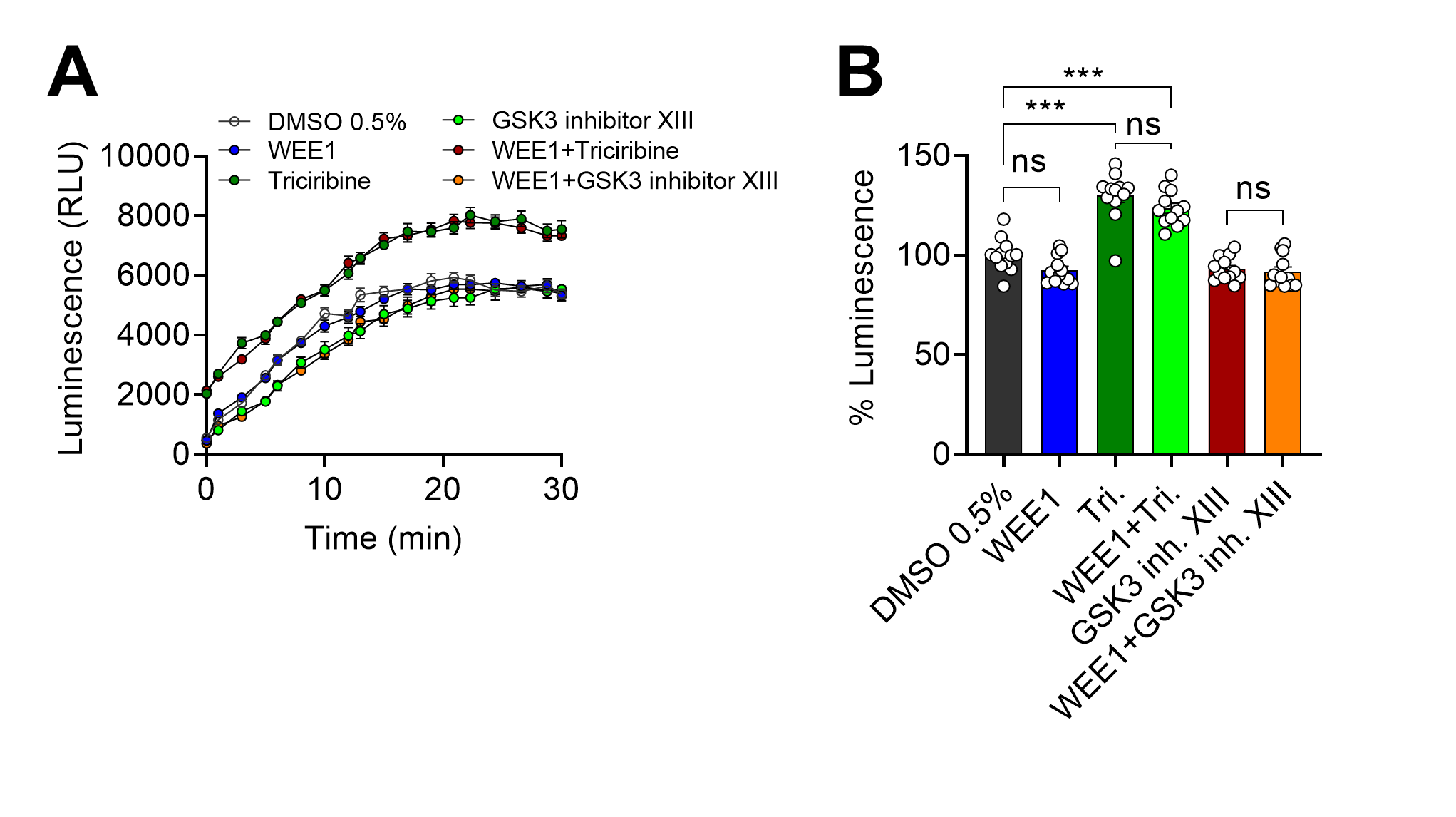


**Supplementary figure S1.** Evaluation of the effects of kinase inhibitors on FGF14Y158A/Nav1.2 complex assembly using the split-luciferase complementation assay (A) Representative real-time luminescence plot for the indicated kinase inhibitors. (B) Representative bar graph to show the effects of WEE1 inhibitor II (15 μM), the AKT inhibitor triciribine (25 μM), and GSK3 inhibitor XIII (30 μM) alone or in the indicated combi-nations on FGF14/Nav1.2 complex assembly. DMSO (0.5%) was used as a vehicle and served as a control. Percentage luminescence (normalized to per plate control wells treated with 0.5% DMSO; n=12 wells per plate). Statistical significance is indicated as ***p<0.0001, ns=non-significant, determined by one-way ANOVA followed by Tukey’s multiple comparisons test. Data are mean ± SEM.


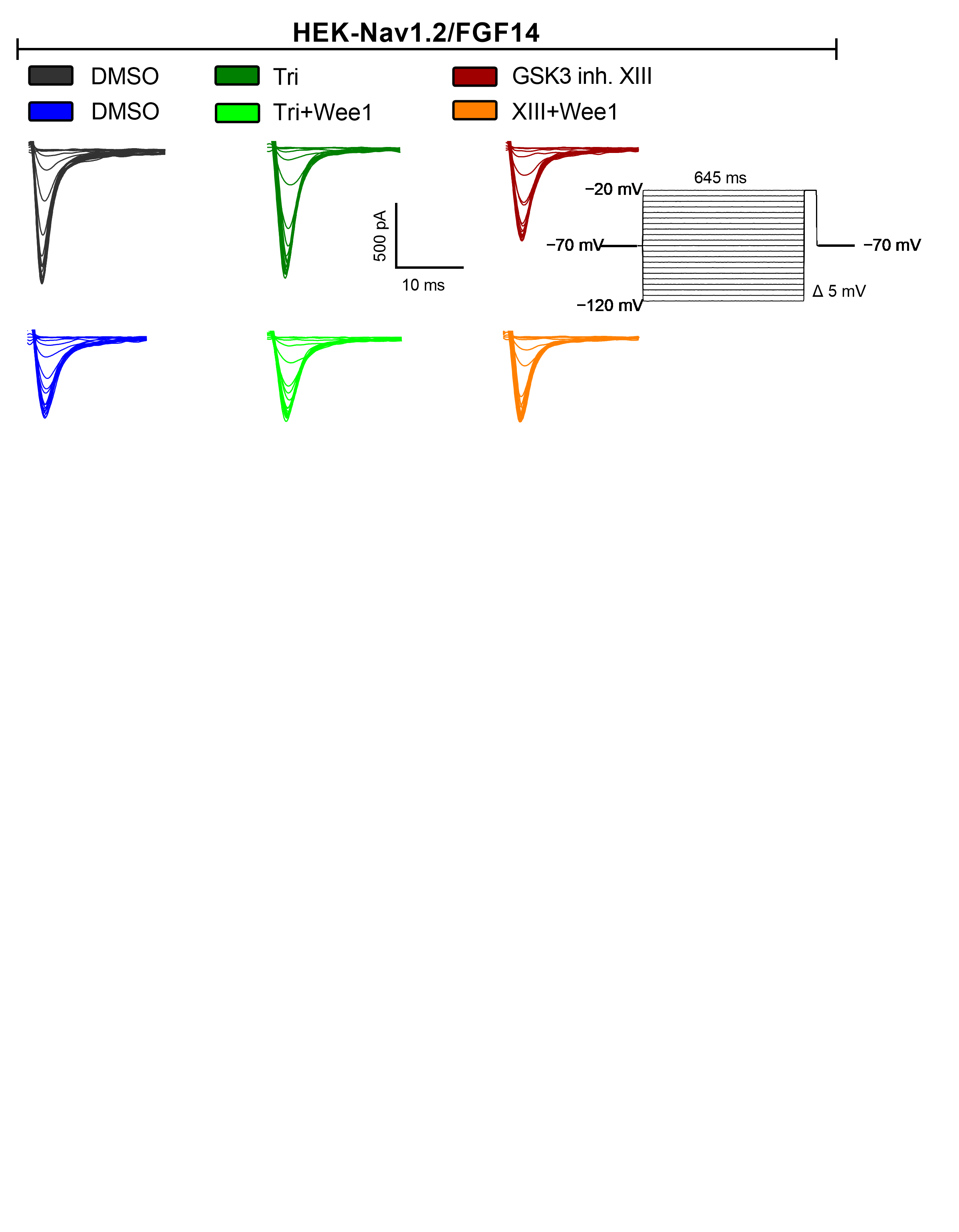


**Supplementary figure S2.** (A) Representative traces of steady-state inactivation were recorded in response to the voltage-clamp protocol depicted on the side in HEK-Nav1.2/FGF14 cells in the presence of the indicated kinase inhibitors. DMSO (0.5%) was used as a vehicle control.
